# Matrix-Encoding Gene Diversity of 624 Influenza A/H3N2 Genomes Does Not Show Association with Impaired Viral Detection by Commercialized qPCR Assays

**DOI:** 10.3390/v14122683

**Published:** 2022-11-30

**Authors:** Lorlane Le Targa, Houmadi Hikmat, Céline Boschi, Bernard La Scola, Philippe Colson

**Affiliations:** 1 IHU Méditerranée Infection, 19-21 Boulevard Jean Moulin, 13005 Marseille, France; 2 Microbes Evolution Phylogeny and Infections (MEPHI), Institut de Recherche pour le Développement (IRD), Aix-Marseille University, 27 Boulevard Jean Moulin, 13005 Marseille, France; 3 Biosellal, 27 Chemin des Peupliers, 69570 Lyon, France; 4 Assistance Publique-Hôpitaux de Marseille (AP-HM), 264 rue Saint-Pierre, 13005 Marseille, France

**Keywords:** influenza A/H3N2 virus, qPCR, diagnosis, matrix gene, M1 gene, genetic diversity

## Abstract

As for the case of SARS-CoV-2, genome sequencing of influenza viruses is of potential interest to raise and address virological issues. Recently, false-negativity of real-time reverse transcription-PCR (qPCR) assays that detect influenza A/H3N2 virus RNA were reported and associated with two mutations (A37T and C161T) in the Matrix-encoding (M1) gene located on viral segment 7. This triggered a national alert in France. The present study sought to assess the association between the presence of these mutations and potential false negative results of influenza A/H3N2 virus RNA detection by commercialized qPCR assays at the clinical virology laboratory of our university hospitals in southern France. This study focused on the genetic diversity in the M1 gene and segment 7 of 624 influenza A/H3N2 virus genomes obtained from respiratory samples having tested qPCR-positive with M1 gene-targeting assays in our clinical virology laboratory. A total of 585 among the 624 influenza A/H3N2 virus genomes (93.7%) were of clade 3C.2a1b.2a.2, and 39 (6.3%) were of clade 3C.2a1b.1a. M1 gene substitutions A37T and C161T were both present in 582 (93.3%) genomes, only of clade 3C.2a1b.2a.2. Substitution A37T was present in 621 (99.5%) genomes. Substitution C161T was present in 585 genomes (93.8%), all of clade 3C.2a1b.2a.2. Moreover, 21 other nucleotide positions were mutated in ≥90% of the genomes. The present study shows that A37T/C and C161T mutations, and other mutations in the M1 gene and segment 7, were widely present in influenza A/H3N2 virus genomes recovered from respiratory samples diagnosed qPCR-positive with commercialized assays.

## 1. Introduction

Genomic surveillance of SARS-CoV-2 has led to a tremendous amount of viral genomes available from multiple countries worldwide during the pandemic due to this virus. It has proved to be powerful in differentiating and classifying viral strains, increasing the understanding of viral evolution, getting a better knowledge of viral genotypic and phenotypic characteristics, allowing to design accurate real-time reverse-transcription PCR (qPCR) systems for the diagnosis of infections and variants, and studying the sensitivity of viral lineages and variants to neutralizing antibodies elicited by past infections or vaccine immunization [1,2]. However, such genome sequencing effort has been far more limited for other respiratory viruses.

The emergence and circulation of influenza A/H3N2 viruses carrying mutations associated with false-negative results of qPCR tests have been recently reported in Denmark [3]. This triggered a national alert in France [4]. These mutations are located in the M1 gene that encodes the viral matrix protein and is located on genome segment 7, which also harbors the M2 gene that encodes a viroporin [5]. The low genetic diversity identified so far for the M1 gene made it a target of several qPCR commercial assays. Nonetheless, Jørgensen et al. correlated false-negative qPCR results, identified by qPCR-positivity with alternative systems, including some targeting other genomic regions, with the presence of mutations in two M1 gene regions targeted by PCR primers [3]. They analyzed eight M1 gene sequences obtained from clinical samples diagnosed as either PCR-positive (*n* = 4) or negative (*n* = 4), plus eight genomes of representatives of circulating strains from the Danish National Reference Center and Surveillance Laboratory for Influenza. Here, we aimed to investigate the presence and prevalence of mutations described by Jørgensen et al. in the M1 gene and segment 7 of influenza A/H3N2 virus genomes obtained from respiratory samples tested by qPCR at our university hospital clinical virology laboratory and the potential association of these mutations with false-negative qPCR results.

## 2. Materials and Methods

The genetic diversity of the M1 gene and segment 7 was analyzed from 624 influenza A/H3N2 genomes (GenBank accession no. OP546682-OP547305 [6,7]) that had been obtained from respiratory samples collected between September 2021 and April 2022 and were sent for diagnosis of respiratory virus infections to our clinical virology laboratory at university hospitals of Marseille, southeastern France. Influenza A virus infections were diagnosed using the FTD Respiratory pathogens 21 (FTD) (Fast Track Diagnosis, Luxembourg), the GeneXpert Xpert Flu/RSV (GeneXpert) (Cepheid, Sunnyvale, CA, USA), or the BioFire FilmArray Respiratory panel 2 plus (BioFire) (Biomérieux, Marcy-l’Etoile, France) assays, which all target the M1 gene (plus two other genes for the GeneXpert assay).

For influenza A virus genome sequencing, viral RNA was extracted from respiratory samples using the EZ1 Virus Mini Kit v2.0 on an EZ1 Advanced XL instrument (Qiagen, Courtaboeuf, France) or the KingFisher Flex system (Thermo Fisher Scientific, Waltham, MA, United States), following manufacturer’s instructions. Then, viral RNA segments were amplified by a procedure adapted from that described by Zhou et al. [8], and next-generation sequencing (NGS) of the amplicons was performed using Illumina technology on a NovaSeq 6000 instrument (Illumina Inc., San Diego, CA, USA), as previously described [9]. Viral genomes were assembled by mapping NGS reads on the genome of the influenza A/H3N2 virus strain A/New York/392/2004 (GenBank Accession no. NC_007366.1-NC_007373.1) with the CLC Genomics workbench v.7 software [10]. 

## 3. Results

**Figure 1:**
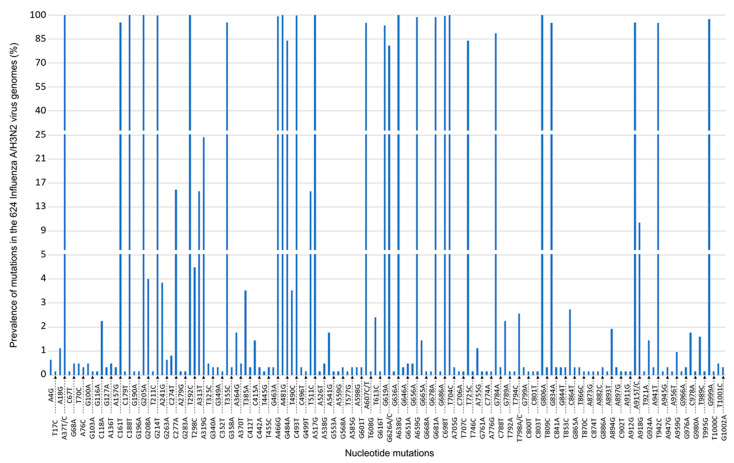
Prevalence of nucleotide mutations in segment 7, including the M1 (matrix) gene of the 624 Influenza A/H3N2 virus genomes analyzed in the present study. The Y-axis is in three parts to better visualize the different levels of prevalence of the mutations.

Among the 624 genomes of the influenza A/H3N2 virus, 585 (93.7%) were of clade 3C.2a1b.2a.2 and 39 (6.3%) of clade 3C.2a1b.1a [11]. The two substitutions A37T and C161T described by Jørgensen et al. [3] within the M1 gene were both present in 582 (93.3%) genomes, only in those of clade 3C.2a1b.2a.2 (Figure 1). 

Nucleotide A37 was mutated in all genomes, substitution A37T being present in 621 (99.5%) genomes and A37C in three genomes obtained from samples collected in December 2021 (*n* = 1) and March 2022 (*n* = 2). Substitution C161T was present in 585 genomes (93.8%), all of clade 3C.2a1b.2a.2. In addition, substitution A76C located in the M1 gene region targeted by the probe of the qPCR system described by Jørgensen et al. [3], was present in two genomes of clade 3C.2a1b.1a. Apart from these mutations, 143 other positions of segment 7 were mutated in ≥1 genome, including 21 that were mutated in ≥90% of the genomes.

The 624 influenza A/H3N2 virus genomes had been obtained from respiratory samples diagnosed by qPCR as positive for influenza A virus RNA. In 616 cases (98.7%), the diagnosis of influenza virus infection was made with the FTD assay, which targets the M1 gene of influenza A viruses but whose performance was not assessed by Jørgensen et al. [3]. In seven cases (1.1%), the diagnosis was made with the GeneXpert assay, which was reported by Jørgensen et al. not to cause false negativity for 6 samples [3]. In one case, the diagnosis was made with the BioFire assay, reported by Jørgensen et al. to cause false negativity for two of five samples [3]. As the 624 respiratory samples tested positive with these qPCR assays, this indicates that mutations A37T/C ± C161T in the M1 gene do not seem to be associated with false negative results in our experience. The mean (±standard deviation) qPCR cycle threshold value (Ct) for these samples with the FTD assay was 25.5 ± 6.9 (range, 19.0–34.0). One respiratory sample tested qPCR-positive with the BioFire assay. The viral genome obtained from this sample was of clade 3C.2a1b.2a.2 and harbored both A37T and A161T in the M1 gene, as well as 25 additional mutations in segment 7. In addition, six nasopharyngeal samples among those available in the biobank for storage at −20 °/−80 °C of our laboratory and exhibiting the highest Ct (range, 29–30) as determined by the FTD assay, were retrospectively tested with the BioFire assay. The genome obtained from these samples was of clade 3C.2a1b.2a.2 in four cases and of clade 3C.2a1b.1a in two cases, and all six genomes harbored mutations A37T and A161T. All these six samples tested influenza A/H3N2 RNA-positive with the BioFire assay, indicating that the presence of mutations A37T and A161T does not seem to be associated with false-negative results using this multiplex qPCR assay. Notably, the six viral genomes also harbored between 17 and 27 other mutations in segment 7.

## 4. Discussion

The present study confirms the presence of both mutations A37T and C161T in the vast majority (99.5%) of the influenza A/H3N2 genomes of clade 3C.2a1b.2a.2 as well as the systematic presence of a mutation at nucleotide 37 for influenza A/H3N2 viruses that circulated since 2021 in Marseille and its geographical area. This study also reports, on a much larger scale than in Jørgensen et al.’s study [3], the majority circulation of clade 3C.2a1b.2a.2 in our geographical region in 2021 and 2022. This viral clade was characterized in 2021 and was the majority influenza A/H3N2 clade during the epidemic that occurred in 2021-2022 in Western developed countries [11]. As the 624 influenza A/H3N2 virus genomes obtained in our laboratory were from respiratory samples diagnosed as influenza A virus-positive by M1 gene targeting-qPCR, mutations A37T and C161T in this gene do not seem to be associated with false negative results in our experience. Nevertheless, the possibility of such qPCR failure or underquantification of viral RNA in the presence of these two mutations, either alone or in combination, or of other mutations in the M1 gene, cannot be excluded. The precise genome regions of the M1 gene targeted by the commercialized qPCR assays are not known, which prevents speculating on the impact of mismatches due to mutations on the performances of detection and quantification of influenza A virus RNA by these assays. Besides, the present study did not directly address whether the recently emerged mutations in the M1 gene might result in the under-quantification of viral RNA; this could rely on the testing of several samples using qPCR assays targeting a single region located either within or outside the segment 7 of the viral genome and harboring or devoid of mutations. To our best knowledge, no country other than France has yet emitted an alert for impaired qPCR performance to detect recently or currently circulating influenza A/H3N2 viruses.

Finally, it is worth noting that the sequencing of the genomes of influenza viruses is currently dramatically underperformed compared to SARS-CoV-2 genomes. According to the GISAID database [12,13], as of 5 October 2022, 141 influenza A/H3N2 virus sequences have been obtained in France from human samples collected since January 2021 and this number is one according to the NCBI Influenza Virus Database sequences [14]. Hence, influenza virus genomic surveillance deserves to be carried out more extensively, in particular, to ensure the performance of commercialized qPCR tests, but more generally in order to draw from it the information that has shown great interest in the context of the genomic surveillance of SARS-CoV-2 infections [1,2].

## Data Availability

Viral genomes analyzed in the present study have been deposited in the NCBI GenBank sequence database under Accession nos. OP546682-OP547305 [6,7].
